# The spinner model for flourishing through movement: a conceptual framework for exploring the interplay between physical literacy, meaningful physical activity, embodiment, and human flourishing

**DOI:** 10.3389/fspor.2026.1819295

**Published:** 2026-07-10

**Authors:** O. Hawes, E. J. Cook, J. Williams, A. M. Chater

**Affiliations:** 1 School of Sports, Psychology and Social Sciences, University of Bedfordshire, Luton, United Kingdom; 2 Institute for Health Research, University of Bedfordshire, Luton, United Kingdom; 3 School of Psychology, University of Birmingham, Dubai, United Arab Emirates; 4 Institute for Sport and Physical Activity Research, University of Bedfordshire, Luton, United Kingdom; 5 Centre for Behaviour Change, University College London, London, United Kingdom

**Keywords:** conceptual framework, delphi, embodiment, human flourishing, meaningful physical activity, physical literacy

## Abstract

**Introduction:**

Emerging evidence suggests that human flourishing is not guaranteed, even in economically developed nations, with substantial disparities reported across populations and contexts. This highlights an urgent need for scalable, context-sensitive approaches that support flourishing and holistic wellbeing. Physical activity represents one such pathway, with growing evidence linking participation to psychological, social, and physical dimensions of flourishing. However, many strategies aimed at increasing physical activity overlook the complexity of behaviour change and the socio-cultural factors shaping movement experiences. As a result, physical activity is often framed as a prescriptive health behaviour rather than a meaningful, intrinsically rewarding experience. This paper presents a conceptual framework that integrates meaningful physical activity, physical literacy, and human flourishing to inform policy, practice, and research aimed at fostering lifelong engagement in movement and wellbeing.

**Methods:**

A Delphi study was conducted with an international panel of experts working across the fields of meaningful physical activity, physical literacy, and human flourishing (N = 8; 75% female). Through iterative rounds of consultation, including focus group workshops and structured evaluation processes, participants provided feedback to refine a preliminary conceptual framework and establish consensus regarding its clarity, relevance, and practical utility.

**Results:**

The Delphi process resulted in a refined framework characterised by enhanced conceptual coherence, stronger alignment with contemporary theory and policy, and greater clarity regarding the interrelationships among meaningful physical activity, physical literacy, and human flourishing. Expert feedback contributed to a more explicit articulation of the mechanisms through which meaningful movement experiences and physical literacy development may support flourishing across the lifespan.

**Discussion:**

The resulting framework provides a theoretically grounded and practice-oriented model for understanding how movement experiences may contribute to human flourishing. By emphasising the quality, meaning, and embodied nature of physical activity, the framework offers guidance for policymakers, public health practitioners, educators, and community organisations seeking to promote sustainable, lifelong engagement in movement and support holistic wellbeing.

## 1 Background

Human flourishing refers to living a life of optimal functioning (113). It extends beyond happiness or absence of distress to encompass multidimensional wellbeing, including physical and mental health, social connectedness, personal growth, purpose, and the development of personal virtues and capabilities (1–3). While wellbeing is often understood as the presence of positive physical, psychological, and social conditions, human flourishing is conceptualised here as the broader process through which individuals come to live well, integrating these dimensions within their everyday lives. Importantly, human flourishing is not treated as an all-encompassing umbrella for positive outcomes, but rather as a dynamic and context-sensitive process, emerging through lived experiences that connect individuals to themselves, others, and their environment (4). This framing maintains conceptual clarity while allowing flexibility across disciplines.

Emerging global evidence suggests that human flourishing is not guaranteed, even in economically developed countries, with significant international disparities reported (5). This highlights the need for scalable, context-sensitive approaches to promote human flourishing, and for frameworks that prioritise the quality of lived and embodied experiences through which human flourishing is developed and sustained.

Physical activity represents a pathway that may contribute to human flourishing, with growing evidence suggesting its role in supporting psychological, social and physical dimensions of human flourishing (6–8). However, direct research linking movement behaviours to human flourishing remains limited (9), and current promotion strategies for physical activity often over-fixate on the quantity of movement behaviours for strategic health targets e.g., (10, 11), rather than the quality of embodied movement experiences for sustained health and holistic wellbeing (12). For some, this leads physical activity to be perceived as a task rather than a fulfilling embodied experience (13), respectively reducing opportunities for human flourishing and long-term engagement in physical activity.

Policy discussions increasingly recognise the importance of integrating quality movement experiences into daily life (14), although challenges remain in translating high-level strategies into effective local implementation (15). Systemic barriers also persist in addressing movement inequalities across communities (16). This paper responds to these gaps by advancing an integrative approach to promoting human flourishing through movement. Drawing on concepts and theories reciprocally connected to human flourishing, this paper positions physical literacy, meaningful physical activity experiences and embodiment as key conceptual foundations for rethinking the value of movement and supporting sustained human flourishing.

Physical literacy is rooted in a holistic view of the person, emphasising that it is not just about physical skills, but also about the individual's unique motivation, confidence, knowledge and understanding to engage in physical activity for life (17). This philosophical foundation is built on monism (18), existentialism (19), and phenomenology (20, 21), and provides prospects to develop embodied potential, growth, fulfilment, and individual virtues, which are crucial for human flourishing (114). However, there is limited research on the application of physical literacy for human flourishing (22), underscoring a need to explore how the concepts may be reciprocally interconnected.

Meaningful physical activity emphasises the personal significance, inherent enjoyment, and holistic enrichment of movement (23). It views physical activity as a fundamental expression of the self, involving individual values, goals and dispositions (24–26). It also consists experiences that are socially interactive, positively challenging, fun, personally relevant, and allow for the development of motor competencies (23). Previous research have summarised meaningful physical activity experiences to contribute to overall wellbeing (27). Pohlmeyer (28) argued that the enablement of pleasurable and meaningful experiences in daily life is promising for promoting moments of human flourishing, although this relationship is underexplored.

While physical activity is often associated with positive outcomes, contemporary embodied learning research suggests that its effects are contingent on the degree to which movement is meaningful, contextually situated, and experientially integrated (29–31). Conversely, an embodied perspective also recognises that movement does not universally promote human flourishing. Physical activity can be externally imposed (32), misaligned with individual capabilities (33, 34), or experienced as stressful or meaningless (35, 36), and may therefore have limited or even adverse effects on wellbeing. This suggests that the value of physical activity and movement lies not solely in its quantity, but in the nature of the embodied experience it affords. Integrating this perspective allows for a more nuanced understanding of how physical literacy and meaningful physical activity interact to support human flourishing.

This position may be supported by the work of Myrto Mavilidi, which demonstrate that movement-based approaches are most effective when they are meaningfully integrated with the activity (37). Mavilidi et al. (37) further demonstrate that the effectiveness of movement depends on both its relevance to the activity and its level of integration, with the strongest effects observed when movements are both meaningful and embedded within the activity. Although developed within educational contexts, the integration of cognition and cognitive load theory proposed by Zou et al. (115) offers a useful conceptual lens for understanding movement more broadly. Their work suggests that any bodily activity is more effective when it aligns with task-relevant processes and does not introduce unnecessary cognitive demands. Extending this perspective to physical activity, it may be argued that movement is more likely to support engagement and human flourishing when it is experientially coherent and aligned with individual goals and capacities, rather than when it is perceived as effortful, imposed, or disconnected.

Similarly, Zhang et al. (116) propose a dual-process framework, distinguishing between domain-general mechanisms (e.g., arousal, attention) and domain-specific mechanisms (e.g., task-relevant embodied representations) through which movement influences outcomes. Again, although developed through educational contexts, this distinction is conceptually informative for the present study, as it suggests that movement may operate through broad, general pathways and meaning-making processes. This implies that movement may support aspects of human flourishing through general wellbeing effects, as well as personally meaningful and contextually embedded experiences. Collectively, this body of work emphasises that the effects of movement are selective and context-dependent, reinforcing the need to consider relevance, integration, and experiential quality when examining the relationship between meaningful physical activity, physical literacy, and human flourishing.

Overall, there is a lack of integrative learning that explicitly connects physical literacy, meaningful physical activity, human flourishing, and embodiment within a coherent theoretical and practice-oriented framework. This paper addresses this fragmentation by presenting a conceptual framework that connects meaningful physical activity, physical literacy, and human flourishing, with embodiment centring as the golden thread that ties these concepts together. The framework aims to support policy makers, practitioners, and community stakeholders in promoting quality movement experiences that foster sustainable wellbeing and lifelong engagement in physical activity. The research objective is as follows;

- To refine and provide expert-informed insight to the development of a research-informed preliminary conceptual framework which integrates physical literacy, meaningful physical activity and human flourishing and provide those who target increasing physical activity, health, and happiness with evidence-informed insight to help understand quality movement experiences using the three concepts.

## 2 Method

### 2.1 Research design

The study took place between November and December 2025, incorporating a mixed-methods Delphi design, and using participatory thematic refinement and analysis to develop, refine and provide expert-informed insight to the development of a preliminary conceptual framework (38). The Delphi process consisted three stages; (1) completion of an expert evaluation form, (2) participation in a series of focus group discussions, and (3) repeating the expert evaluation form to generate a final judgement of the frameworks' content. This design enabled an integrated understanding of the perspectives of professionals in the field, ensuring that the final framework was not only evidence-informed, but grounded in practitioner and academic expertise (39).

### 2.2 Participants and recruitment

In this study, participants were invited to come forward for the Delphi rounds, using a purposive criterion-based sampling approach to maintain conceptual consistency, strengthen validity, and allow for more depth in the consensus building process (40). Selection and identification criteria for professionals in this study insisted participants to possess at least five years of work experience in relevant fields (e.g., teachers, experienced researchers, professional representatives, interdisciplinary professionals) and have either practical, teaching or research experience in topics of meaningful physical activity experiences, physical literacy, and/or human flourishing. The five-year threshold aligns with Delphi methodology guidance suggesting that experienced professionals are more likely to provide informed judgments and reduce the risk of uninformed responses influencing consensus development (41, 42). This recruitment strategy ensured dialogue with a range of different professionals from different disciplines to allow for sufficient representation.

Delphi study samples can range from as little as four participants (43) to several thousand, with larger samples proposing difficulty in data collection and management (44). In this study, eight participants agreed to take part in the Delphi process. Although, there was attrition between the focus groups and the final responses. Particularly, there was a loss of participants in some of the rounds due to scheduling conflicts and competing family/work responsibilities, with one of the rounds consisting of three participants. As this sample number falls below the typical sample size for Delphi studies (e.g., as little as four participants (43), the overall validity of the interpretations and findings for that round may have been impacted (45). To reduce risk to validity, pragmatism was incorporated by ensuring that all participants who were unable to attend any of the rounds had the opportunity to attend follow-up interviews and provide written feedback of their thoughts regarding the discussions via email. With email responses, all participants were given the transcript for the discussion and a summary of key themes discussed, and provided input based on these materials.

### 2.3 Materials

**Figure 1:**
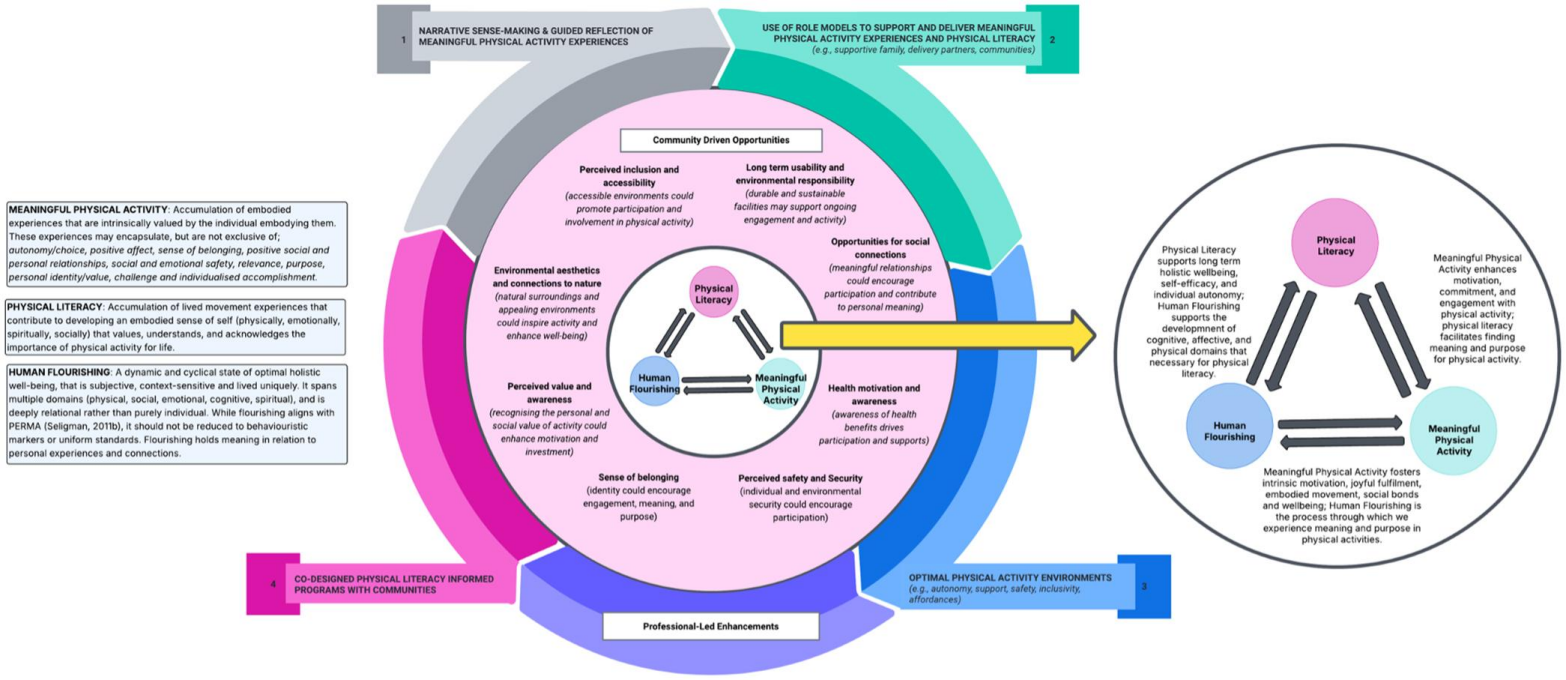
Preliminary conceptual framework.

Participants received a comprehensive evidence pack. This pack included a PDF of a research-informed preliminary conceptual framework (see Figure 1).

The framework in Figure 1 defines meaningful physical activity, physical literacy, and human flourishing (see left hand boxes), demonstrates the interconnectedness between the three concepts (see inner circle), describes some of the influences that may affect how people flourish through movement (see the middle circle), and some of the key practical considerations for enabling human flourishing through movement (outer circle). The framework was developed through an iterative synthesis process informed by multiple data sources. The structural relationships depicted in Figure 1 were generated through thematic integration and mapping of concepts across sources, guided by established approaches to framework development (38).

The central layer of the framework was informed by findings from a systematic integrative review (46), which identified key interconnections between meaningful physical activity, physical literacy, and human flourishing. The key findings of this review were that the three concepts mutually reinforce one another. The primary mechanisms responsible for this interconnection included shared core features and foundational qualities, such as enjoyment, social connection, challenge and personal relevance. Additionally, it was summarised that individual experiences of meaningful physical activity trigger developmental cascades that support skill development, competence, confidence for physical literacy and overall human flourishing. However the review also highlighted that human flourishing may not occur in all physical activity experiences due to individualistic tendencies, environmental factors, and temporal factors. A summary of these findings was provided to participants to facilitate in the evaluation of the preliminary framework.

The middle layer of the framework was informed by the MoveScape study (47), which explored how individuals experience meaningful physical activity within community environments and the factors that may influence meaningful physical activity, physical literacy and human flourishing. The study identified contextual and environmental influences on engagement and wellbeing, such as accessibility, safety, social connection, and sense of belonging. This supported the inclusion of broader influences within the framework. Participants were provided with full access to this article when evaluating the preliminary framework, and a summary of how the findings link to the framework development.

Insights from semi-structured interviews with 26 professionals in relevant fields were also incorporated to inform the preliminary conceptual framework. Due to the unpublished status of this work, only aggregated themes and interpretive summaries were shared with participants, rather than raw data or full transcripts. Ethical approval for the study was obtained from the University of Bedfordshire, and participants consented to the use of anonymised data for research purposes. This approach ensured transparency while protecting the integrity and future publication of the study. The themes and interpretations of these interviews informed the central, middle, and outer layer of the framework. The findings from the interviews demonstrated how professionals saw the concepts as interconnected and how this interconnection may work in practice. Specifically, professionals emphasised the importance of narrative sense-making, guided reflection, role models, co-designed programmes, and supportive environments for enabling human flourishing through movement, physical literacy, and meaningful physical activity experiences. These practice-oriented factors informed the outer layer of the framework, representing key considerations for implementation. Taken together, these data sources enabled triangulation and strengthened the conceptual and empirical grounding of the framework.

Participants were also provided with an expert evaluation form to assess the overall credibility of the framework (Supplementary File S1). The expert evaluation form was adapted from the Maturity Model Domain Expert Evaluation Form developed by (48), which assesses a model's constructs and instruments by identifying strengths, weaknesses, and priorities for improvement. The questions used in the form were adapted to align with the context of the study. This form was chosen as an evaluation tool as it helped to directly assess the structural and conceptual integrity of the framework, rather than focusing merely on measurement properties or testing for specific outcomes.

### 2.4 Procedure

**Figure 2:**
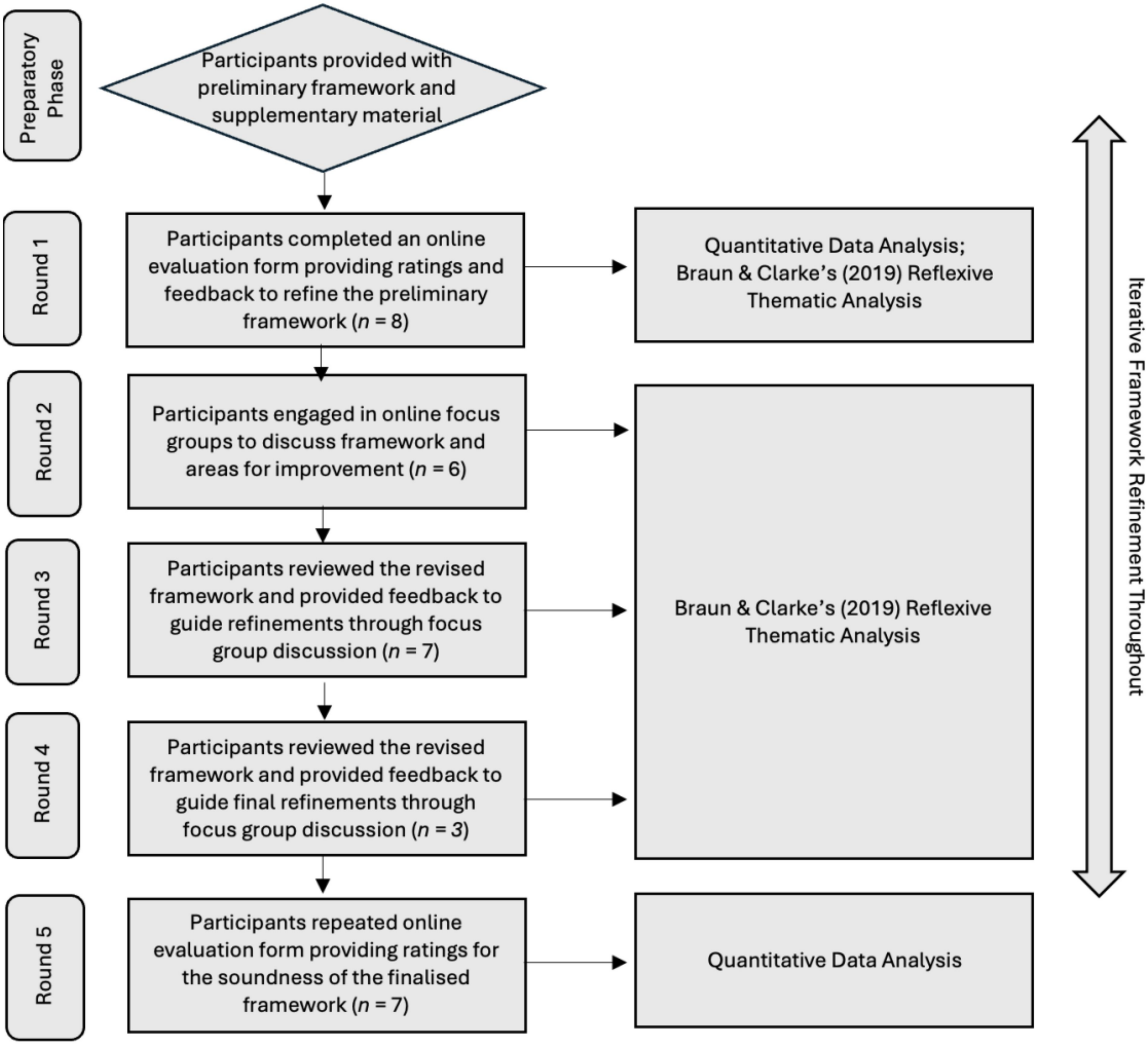
Visual presentation of the stages of the delphi process.

The study comprised 5 rounds (see Figure 2) falling in line with Delphi study recommendations of 3–5 rounds (49). The number of rounds were not set in stone, but rather the researcher (OH) and participants continued the rounds until iterative feedback no longer resulted in substantive modifications to the framework, indicating that consensus stability and data saturation had been met (50, 51). The evaluation forms somewhat informed the number of rounds, as the output from these forms determined the overall soundness of the refined framework; once the framework was considered “sound”, with ≥75% consensus agreement (52), the rounds were no longer required. In the early rounds, the researcher (OH) avoided rigid question-and-answer formats, adopting a conversational approach with open-ended questions to encourage deeper reflection (117). As the Delphi progressed, greater emphasis was placed on consensus building to support final decision making. This ensured that the discussion was grounded in expert-generated insights while allowing for contextualisation of participant thoughts, opinions, and critical feedback.

In the first round, participants reviewed the preliminary conceptual framework and rated statements on a five-point scale, from Strongly Disagree (1) to Strongly Agree (5), relating to sufficiency (e.g., Does framework provide sufficient information to understand meaningful physical activity experiences, physical literacy, and human flourishing), accuracy (e.g., is the information in the developed framework accurate and can it be supported by comprehensive evidence), relevance (e.g., does the framework cover the essential elements related to meaningful physical activity experiences, physical literacy, and human flourishing), comprehensiveness (e.g., does the developed framework comprehensively cover components essential for promoting human flourishing), understandability (e.g., is the language and terminology used clear and understandable), ease of use (e.g., is the developed framework user friendly?), and usefulness/practicality (e.g., The developed framework is practical for use advancing research, policy, or practice in the field). Participants also responded to a series of open-ended questions that invited further comments and suggestions. This included: “What additional information could improve the framework's sufficiency?” and “How could the framework better address the interconnectedness between meaningful physical activity experiences, physical literacy, and human flourishing?”. The form was piloted by a staff member at the University of Bedfordshire to enhance clarity, validity, and the overall data collection process (53, 54). The evaluation form took approximately 30 minutes to complete. This form was repeated in the fifth round, however, participants were not required to respond to open ended questions, as consensus had already been reach in rounds two to four.

In rounds two to four, focus group discussions took place online via Microsoft Teams (55), and drew from participatory action research and co-design approaches where participants collaboratively provided expert-informed insight, critique, and adaption through consensus and refinement (56). An iterative topic guide was provided as an aide memoire to inform the discussions (57, 58). Several questions in the guide were informed by principles of conceptual framework evaluation, emphasising conceptual clarity, practical relevance and language accessibility (59, 60). Indicative questions included; “what were your first impressions of the revised framework, what stood out to you?”, “How do you feel about the language, tone, and structure of the revised framework compared to the initial version?”, and “what specific changes or additions do we agree would enhance the framework?”.

### 2.5 Ethical considerations

Ethical Approval was sought from the University of Bedfordshire (IASR_0125) and the study adhered to the principles outlined in the BPS code of Code of Ethics and Conduct 2021 (61). Participants and any other persons mentioned in the Delphi process were kept confidential and names were pseudo-anonymised for confidentiality. Participants had the right to withdraw their consent and data up to 30 days post data collection, at which point the data was anonymised and shared. Data was stored on a password protected computer and processed in line with the University of Bedfordshire's GDPR policies. Only the researcher and the participants were present in the discussions. Field notes were taken during each focus group to ensure the researcher (OH) captured immediate reflections that could inform data analysis and interpretation.

## 3 Data analysis

For round one and five, an average score was generated for how strongly participants agreed that the framework was sufficient, accurate, relevant, comprehensive, understandable, easy to use, useful, and practical. For scores below 4, it was deemed that no consensus was reached (62). For “relevance”, an overall score was generated by averaging the responses of the two questions corresponding to that construct. A consensus was gathered for the overall soundness of the preliminary conceptual framework using the a Delphi consensus threshold of ≥75% agreement (52). This was calculated by gathering the average consensus agreement of all constructs (sufficiency, accuracy, relevance, comprehensiveness, understandability, easy to use, practicality and usefulness). If the overall participant consensus of soundness of the framework was below 75%, the framework was not deemed sound, and therefore further Delphi rounds consisting refinement were required.

For the qualitative feedback data within the evaluation forms, structured framework analysis (63, 64) was used, combining inductive thematic identification with deductive coding against predefined evaluation constructs (sufficiency, accuracy, relevance, comprehensiveness, understandability, ease of use, usefulness, and practicality). This allowed for transparent links to framework refinements across Delphi rounds.

For rounds two to four, Braun and Clarke's (65) reflexive thematic analysis was conducted using inductive coding to explore for key themes related to how the framework could be improved. The six-phase iterative guide for the analysis included becoming familiar with the data, generating initial codes (on the interpretative level), searching for themes, reviewing themes, defining themes, writing up the themes, and providing an analytic interpretation (65). This process ensured a consistent, trustworthy and rigorous approach to the analysis (66), allowing for each dataset to speak for itself (67). A series of recommendations and action points were developed from the codes and emerging themes to help inform how the preliminary framework may be revised. The researcher (OH) also produced descriptive statistics from quantitative data of expert evaluation forms. All data analysis was managed in Microsoft Excel (68), enabling transparent cross-round comparison, systematic tracking of consensus, and efficient management of structured datasets.

To enhance the overall validity and robustness of the findings, several strategies were employed. Although the Delphi panel size was modest and decreased across rounds, this is consistent with Delphi methodology, where emphasis is placed on the informed judgement of expert participants rather than sample size alone (69). Validity was also strengthened through the use of established consensus thresholds (52), structured and transparent analytical frameworks, and the integration of both quantitative and qualitative data across rounds. The iterative nature of the Delphi process allowed participants to reflect on and refine their responses in light of group feedback, supporting the stability and credibility of the findings. To reduce risks to validity associated with participant attrition, a pragmatic approach was adopted, whereby methodological flexibility was used to retain expert input across Delphi rounds (70). Participants who were unable to attend scheduled sessions were offered alternative means of contribution, including follow-up interviews and written feedback via email. This approach ensured continuity of expert perspectives, minimised data loss, and reduced the risk of bias associated with declining participation, thereby supporting the overall credibility and completeness of the findings.

## 4 Results

### 4.1 Participant demographic characteristics

Table 1 summarises participant demographics. Of the eight participants, one was aged 25–34 years, four were 35–44 years, and three were 45–54 years. Six identified as female and two as male. Most (75%) held a postgraduate qualification, including four with a PhD. Professional roles spanned academia and applied contexts, including professors, researchers, lecturers, directors, practitioners, and consultants. Many participants held multiple roles (e.g., lecturer and researcher, or researcher and consultant). Overall, the panel reflected a diverse range of academic, professional, and applied expertise across education, community sport, and consultancy settings. Participants were highly experienced in areas related to meaningful physical activity, physical literacy, and/or human flourishing, with most reporting over 15 years of professional experience. The sample was internationally diverse, comprising participants from India (*n* = 3), the UK (*n* = 4), and the USA (*n* = 1). Given this breadth of expertise across sectors and countries, the panel aligns with established Delphi guidance that prioritises the quality and relevance of expertise over large sample sizes (71).

**Table 1:** Participant descriptives.

Name	Age Range	Gender	Highest Level of Education	Professional Role/Job Title	Years of Experience	Sector or Setting	Country/Region of Practice
Anya	45–54	Female	PhD	Director	30	Educational Global Consultant, Curriculum Developer & Trainer	India
Clara	35–44	Female	PhD	Professor of Physical Literacy and Physical Education	15+ Years	Higher Education, Policy	UK
Grace	35–44	Female	MSc	Education Subject Area Lead- Higher Education	5+ Years	Education	UK
Jordan	35–44	Female		Activities Coordinator & Exercise Physiologist	25+ Years	Community Sport and Physical Activity	USA, Colorado
Liam	25–34	Male	BA Hons	Lead Sports Manager at Kids Planet Day Nurseries	10	Education	UK
Rohan	35–44	Male	PhD	Managing Director	15	Education	India
Sarah	45–54	Female	Masters	Lecturer & Researcher	30+ Years	Higher Education, teacher education	UK
Sunita	45–54 years	Female	PhD	Researcher & Consultant	25 + years	Education and Community	India

### 4.2 Thematic findings

The Delphi findings are presented on a round-by-round basis to reflect the method's iterative design. This structure demonstrates how expert feedback was incorporated, how consensus evolved across rounds, and how the conceptual framework was progressively refined in response to participant ratings and comments. Results for each round are outlined below.

#### 4.2.1 Round One

Table 2 presents the evaluation of the preliminary conceptual framework. Overall, while participants (*n* = 8) generally agreed that the framework was relevant and conceptually accurate, overall consensus for soundness was 68.75%, falling below the commonly applied Delphi threshold of ≥75% agreement (52). This indicated that the framework was not yet sufficiently robust in demonstrating the interconnectedness between meaningful physical activity, physical literacy, and human flourishing, nor in functioning as a practical tool to enhance human flourishing through movement. Qualitative feedback reinforced the need for refinement, particularly regarding practicality and usefulness, with 88% of participants identifying this as an area for improvement.

**Table 2:** Evaluation results of the preliminary conceptual framework.

Framework Domains	Average Score	Agreement on Soundness	Need for Improvement (%)	Example Quote Illustrating Areas for Improvement
Sufficiency	3.9	No consensus reached	63%	“No more details. It could be simplified, there's a lot of information in the framework.” (Jordan) “Clarification and consolidation for the interconnectedness and how the terms interrelate, would be beneficial.” (Grace)
Relevance	4.25	Yes	63%	“I think the problem you have is in presenting them as discrete concepts (silos) instead you should overlap the concepts identifying what is similar and different using a concept analysis approach. You will then find many of the core similarities are in the overlapping area of the Venn-diagram” (Clara)
Accuracy	4	Yes	50%	“I think the four-point Professional-Led Enhancements would benefit from further context and supporting evidence to ensure these are interpreted and accurately understood for their relevance, impact and application to support the interconnectedness of the three elements. I think it is important to ensure the four points are clear to illustrate why these are important, how these are facilitated in practice, and how they are linked to Community Driven Opportunities” (Grace)
Comprehensiveness	3.8	No consensus reached	63%	“A brief elaboration on the psychological and affective dimensions that underpin human flourishing (e.g., sense of purpose, vitality, and self-fulfilment) would enhance the framework's comprehensiveness and applicability across educational and community contexts.” (Anya)
Understandability	3.6	No consensus reached	63%	“There is a lot of text and that might intimidate some users (non-native English speaking audience). Maybe if it can be presented in separate layers as well? And then the entire thing be brought together..so wheel 1, wheel 2 and wheel 3.” (Sunita)
Ease of Use	3.4	No consensus reached	38%	“User-friendliness could be enhanced by including:.. A glossary of core terms, supported by examples from reviewed studies, to help educators and practitioners quickly apply the framework… [and] Implementation scenarios or “practice lenses” (e.g., school PE, community wellness programs, rehabilitation).” (Anya)
Practicality/Usefulness	3.9	No consensus reached	88%	“You must first ask the question what is the framework going to be used for? And by whom this will then help you to refine the framework for usability. Who will use it and for what?” (Clara) “It's comprehensive. Outside of an academic setting, I'm not clear how leaders of physical activity experiences would know how to apply each of the components to their sessions and ensure they are meeting all the areas within the framework.” (Jordan)

Concerns about sufficiency and comprehensiveness centred on limited or unclear definitions, especially of physical literacy and human flourishing. Participants called for clearer operational definitions, broader conceptual coverage (beyond psychological interpretations of human flourishing), and inclusion of key terms familiar to academics and practitioners. Several also noted that the framework appeared visually overcrowded and conceptually complex.

Although relevance was rated positively, participants felt the visual representation treated the three constructs as siloed rather than integrated, suggesting alternative formats (e.g., overlapping or layered models) to better illustrate conceptual intersections. Accuracy was also questioned in the middle and outer layers, particularly regarding “Professional-Led Enhancements” and “Community-Driven Opportunities” (see Figure 1) which required clearer explanations and stronger evidential grounding.

Understandability and ease of use were hindered by dense text, technical terminology, and limited contextual examples. Suggestions included simplified language, clearer visual design, glossaries of key terms, and applied scenarios. Overall, while the framework was viewed as conceptually promising, participants emphasised the need for structural simplification, clearer integration, and stronger translation into practice to enhance its usability and methodological soundness.

#### 4.2.2 Round Two

Participants (*n* = 6) highlighted the need to clarify the framework's purpose, utility, and philosophical positioning. The framework should communicate why it exists, who it is for, and how it can be applied across contexts. Several participants noted the importance of maintaining a consistent conceptual lens across the three constructs. The definition of human flourishing was perceived as overly psychological, with 63% of participants requesting clearer and more accessible explanations of key concepts. Suggestions included framing human flourishing as an embodied and relational lived experience rather than solely a psychological state, with one participant noting the need for operational clarity for broader audiences:

“Human flourishing may need a brief operational definition for practitioners outside psychology to ensure shared understanding.” (Rohan, Managing Director of Sports Education Company, India).

Overall, feedback supported representing the framework as dynamic, interconnected, and non-linear. Participants criticised the preliminary visual structure, noting that it treated meaningful physical activity, physical literacy, and human flourishing as discrete constructs. Alternative representations such as overlapping or Venn-style models were suggested to better reflect conceptual integration. One participant explained;

“I don’t think there is linearity… you need to show similarities and crossovers.” (Sunita, Researcher and Consultant for Educational Establishments and Communities in India).

Metaphorical explanations were also proposed to illustrate generative relationships between concepts, emphasising fluidity and contextual emergence. A strong theme was the need for a person-centred framework that supports interpretation rather than prescription. Participants recommended simplifying language, reducing text density, and providing glossaries and examples. As one participant stated;

“Doing less will get you more… the framework should invite conversation” (Clara, Professor of Physical Literacy and Physical Education, Founder of Physical Education Organisation based in the UK).

**Figure 3:**
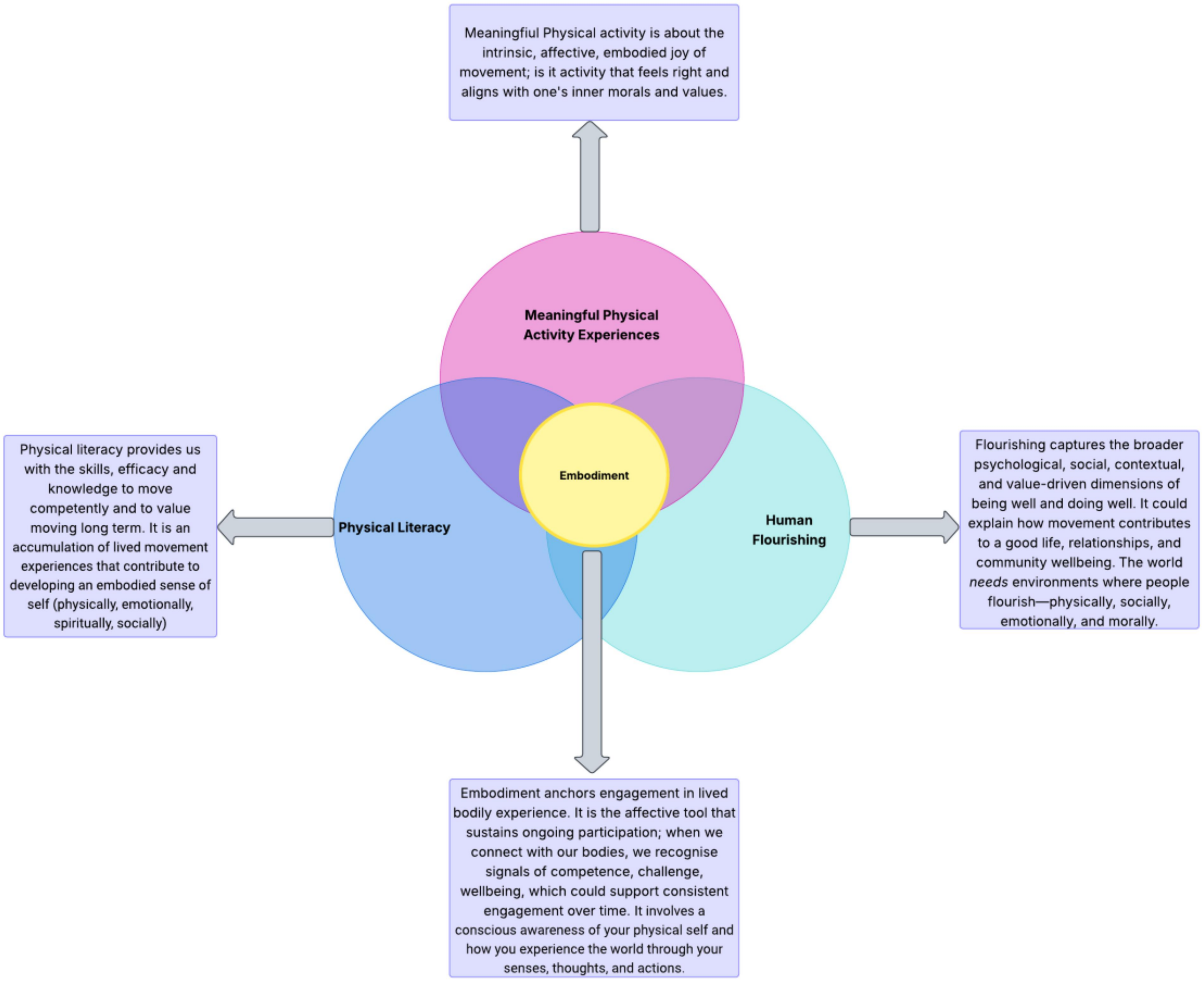
Framework refinement.

Feedback further suggested stripping the framework back to focus on core theoretical intersections while allowing vertical depth for academic, policy, and practical applications. Embodiment was identified as a central integrating principle. Consequently, the framework was revised to reflect the dynamic, emergent relationships and the notion of becoming through movement (see Figure 3).

#### 4.2.3 Round three

Round three focused on evaluating the revised framework, improving visual representation, and finalising conceptual definitions. Focus group participants (*n* = 6) agreed that refinement should begin with macro-level structure, particularly visual representation, before clarifying micro-level conceptual meanings. The group emphasised that the framework should present interconnectedness first, followed by detailed textual explanation. As Clara noted, understanding the relationships between concepts would help determine the nuance of each construct;

“Determining the relationship would then also determine the nuance of each of the terms, because there will be some commonalities that we could put in every single box” (Clara, Professor of Physical Literacy and Physical Education, Founder of Physical Education Organisation based in the UK).

**Figure 4:**
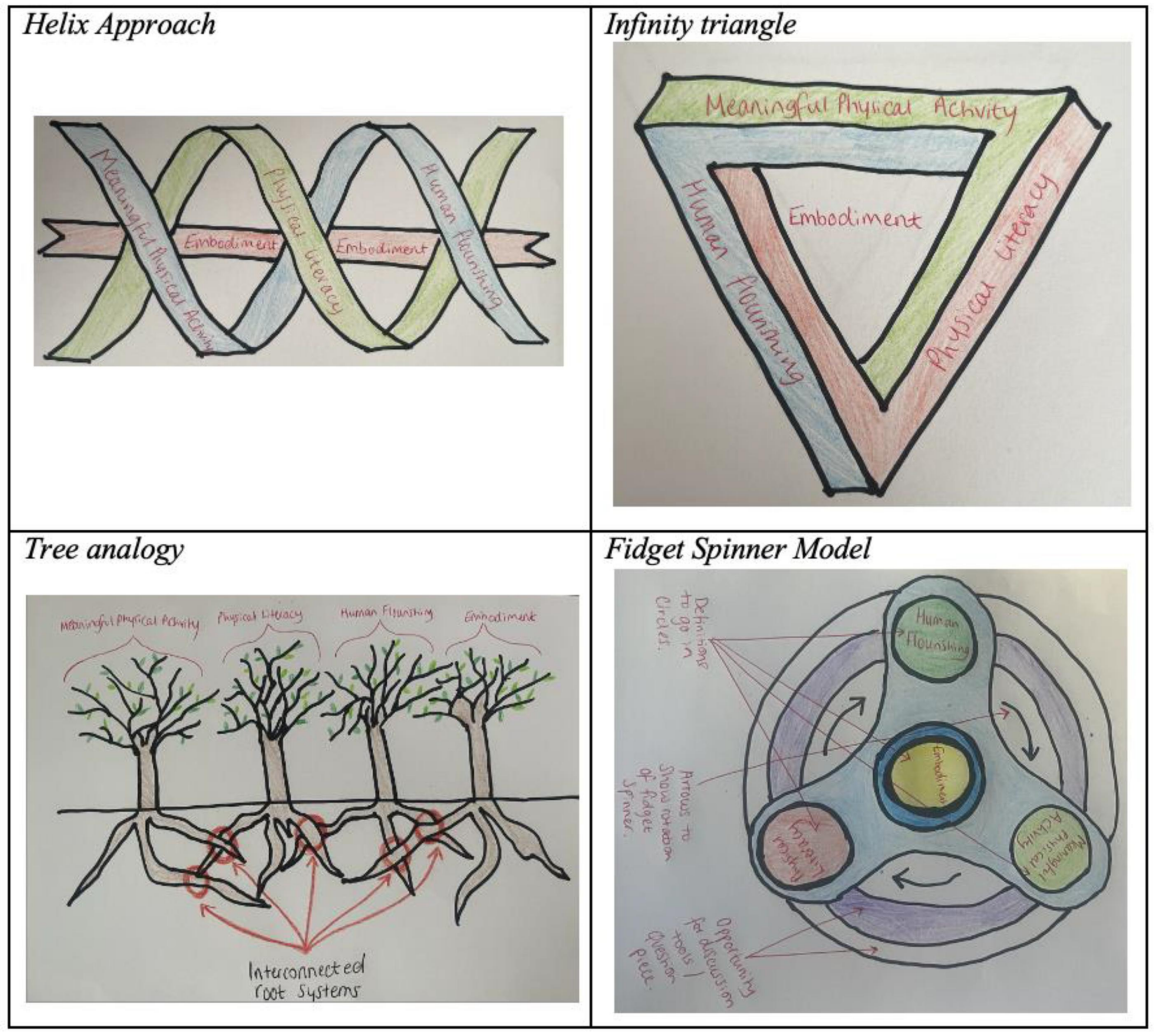
Illustration of discussed models.

Participants questioned whether a Venn diagram was the most effective representation, as it implied partial rather than holistic integration. Alternative dynamic models were discussed, including helix, infinity triangle, a tree analogy, and fidget-spinner approaches (see Figure 4). The helix and infinity models were considered but raised concerns regarding hierarchy and structural interpretation. Metaphorical representations such as tree or rhizomatic structures were viewed positively for illustrating generative, evolving relationships.

Consensus was reached on adopting a fidget-spinner-style visualisation, as it better represented continuous interaction between concepts and movement within a unified system. Participants agreed this model supported non-static, emergent relationships and allowed for interpretive discussion points. The framework was therefore redesigned to emphasise dynamic interconnectedness rather than siloed constructs.

**Figure 5:**
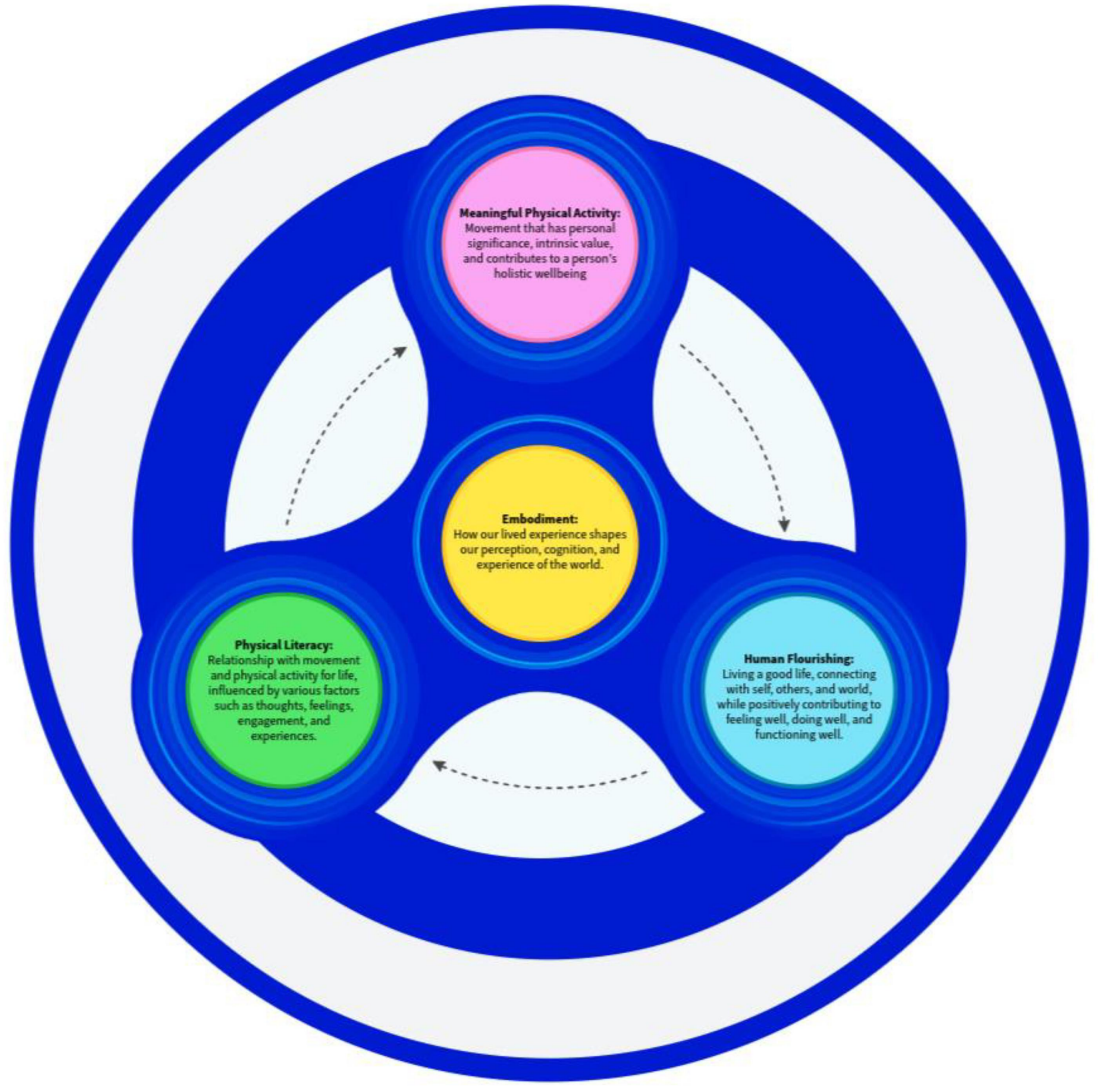
Refinement of conceptual framework in round three.

Conceptual definitions were refined across meaningful physical activity, physical literacy, and human flourishing. Meaningful physical activity was simplified to “movement that has personal significance, intrinsic value and contributes to a person's holistic well-being”. Human flourishing was defined as “living a good life, connecting with self, others, and world, while positively contributing to feeling well, doing well, and functioning well”. Physical literacy was reframed as “Relationship with movement and physical activity for life, influenced by various factors such as thoughts, feelings, engagement, and experiences”. Embodiment emerged as the central integrating principle, described as the “golden thread” linking all three constructs. The revised framework in Figure 5 emphasises person-centred interpretation, accessibility, and contextual adaptability.

#### 4.2.4 Round four

This focus group continued to refine the framework, particularly the definition of embodiment and the design of a reflection/discussion tool to extend the conceptual explanation. Three participants (Anya, Grace, and Liam) attended (*n* = 3), with additional feedback collected via email from other participants who were unable to attend.

The final embodiment definition was refined through eight iterative modifications. The agreed definition was: “A lived way of engaging with and making sense of movement in the world, as it is (re)shaped with every interaction”. Participants emphasised simplicity, accessibility, and alignment with physical activity contexts. Embodiment was conceptualised as integrating lived, felt, and expressed experience, capturing the unity of body and experience within a monist perspective. The term (re)shaping was included to reflect the dynamic influence of movement experiences across contexts and time.

The framework's reflection tool was designed using an “onion” structure, where outer layers represent broad interpretive questions and inner layers contain more focused prompts exploring interconnectedness. Bidirectional arrows were added to illustrate reciprocal relationships between meaningful physical activity, physical literacy, and human flourishing, reinforcing a non-linear, dynamic system. Three guiding questions were included to encourage user interpretation; How do meaningful physical activity experiences and physical literacy interact? How do meaningful physical activity experiences and human flourishing interact? And, how do physical literacy and human flourishing interact? These questions help to support a person-centred, adaptable framework. The reader is encouraged to look at the definitions of each concept, and then critically self-reflect on how each concept relates in their context, and how the relationship between concepts interconnect based on their context.

**Figure 6:**
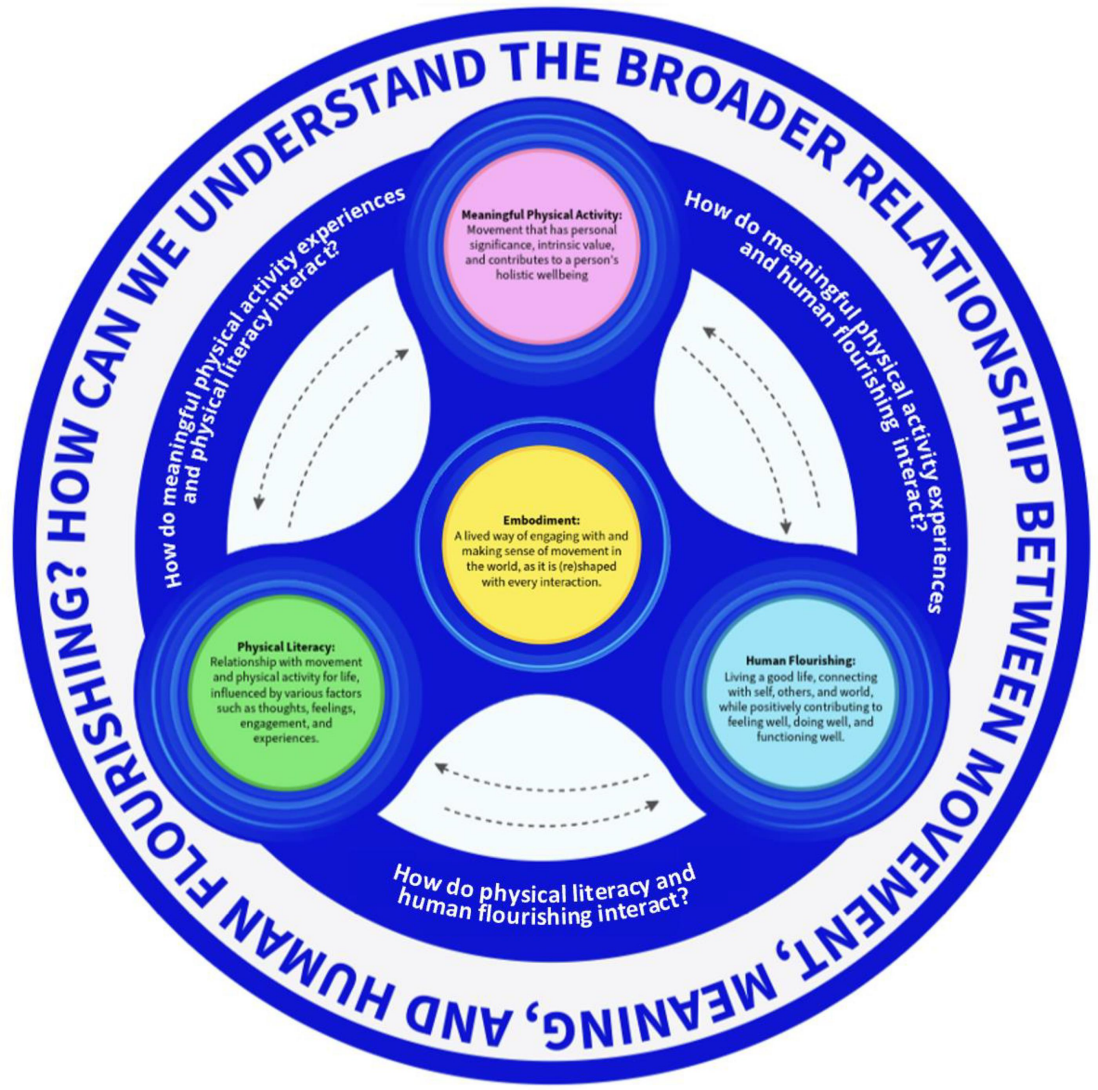
Conceptual spinner model integrating physical literacy, meaningful physical activity, and human flourishing. Note. The model illustrates the dynamic, bidirectional, and non-linear relationships between the three core constructs. Physical literacy represents the individual's capacity to engage in movement through motivation, confidence, competence, and understanding. Meaningful physical activity reflects the subjective value, enjoyment, and personal relevance attributed to movement experiences. Human flourishing is conceptualised as a dynamic and embodied process of living well, evidenced through multidimensional wellbeing outcomes. The arrows indicate reciprocal and evolving interactions between constructs, emphasising that no single component operates in isolation. Embodiment is positioned as the central integrative mechanism (the “golden thread”), through which movement is experienced, interpreted, and situated within individual and contextual realities. The spinner design reflects the fluid, context-sensitive, and continuously evolving nature of these interconnections, allowing for multiple entry points depending on the individual, setting, or purpose of application.

The framework was finalised as the “Spinner Model for Flourishing through Movement” (See Figure 6). Regardless of where an individual enters in the framework, whether that be taking a physical literacy approach, exploring their meaningful physical activity experiences, or delving into how they may reach human flourishing through movement; if they learn how to develop in one area, the fidget spinner will consequently begin to move, and other areas will correspondingly move with it, visually demonstrating a mutual reinforcement and interplay between the concepts that can be easily understood and used in research, policy or practice. A fidget spinner can move at different speeds, and in different directions. This provides a metaphorical explanation for the three concepts in that their progression is entirely subjective and context dependent. By allowing for a framework that is flexible and open to interpretation, it becomes easier for the user to understand how concepts might work together in their own circumstances and settings. Nevertheless, by placing meaningful physical activity, physical literacy, embodiment, and human flourishing within a system (i.e., the fidget spinner), the framework presents the concepts as separate parts to one whole system, thereby aligning with holism, a core philosophical underpinning of all three concepts.

#### 4.2.5 Round five

In the final round, seven participants (Grace, Sarah, Rohan, Liam, Clara, Anya, and Sunita) evaluated the overall soundness of the conceptual framework across seven domains in the expert evaluation form. Percentage agreement was calculated as the proportion of ratings scoring 4 or 5 on a five-point Likert scale. Table 3 presents detailed domain scores. Results demonstrated strong improvement following iterative refinement, with agreement exceeding 85% across all domains. The overall consensus on framework soundness was 94.6%.

**Table 3:** Final evaluation results for the conceptual framework.

Core Domains for Framework	Average Score	Agreement on Framework Soundness
Sufficiency	4.4	85.7%
Relevance	4.7	100%
Accuracy	4.7	100%
Comprehensiveness	4.3	85.7%
Understandability	4.4	100%
Ease of Use	4.1	100%
Practicality/Usefulness	4.3	85.7%

Using the commonly applied Delphi consensus threshold of ≥75% agreement (52), consensus was achieved for all domains, indicating strong panel agreement. The findings suggest the framework effectively represents the synergy between meaningful physical activity, physical literacy, and human flourishing. The framework is therefore considered a credible tool for supporting research, policy, and practice aimed at facilitating human flourishing through movement.

## 5 Discussion

This study aimed to refine and provide expert-informed insight to the development of a preliminary conceptual framework that integrates physical literacy, meaningful physical activity, and human flourishing, providing stakeholders with insight into how these concepts interconnect to support quality movement experiences. The study employed iterative reflection and consensus-building through focus groups and expert evaluation forms to confirm the conceptual, methodological, and practical soundness of the framework. Across Delphi rounds, the framework evolved from a complex, exhaustive structure to a more flexible, dynamic, and emergent model aligned with contemporary research, policy, and practice priorities. The final framework was considered theoretically and methodologically robust by professionals across research, policy, and applied practice contexts.

Early participant feedback highlighted that the preliminary framework was overly complex, visually crowded, and difficult to interpret. Participants recommended simplification to enhance accessibility and usability across multiple professional levels. This aligns with conceptual framework design principles, which emphasise clarity and simplified representation of phenomena (72). Participants also stressed the need for practical relevance and stronger translation between research and practice. Conceptual frameworks should guide knowledge organisation and support implementation into policy and practice contexts (73). Consequently, the framework was redesigned to prioritise both theoretical coherence and practical utility.

The final conceptual integration for the framework is demonstrated through a Spinner Model, incorporating a visual representation and discussion tool around the interconnections between meaningful physical activity, physical literacy and human flourishing. Spinner-style models facilitate multi-stakeholder knowledge transfer and promote innovation rather than fixed solutions (74); the design enables multiple entry points, thereby supporting holism and reinforcing conceptual synergy. The arrows in the Spinner Model represent bidirectional and dynamic interconnections between meaningful physical activity, physical literacy, and human flourishing, illustrating the mechanisms through which each concept can influence and reinforce the others.

Participants' perspectives on the definitions of each concept were generally consistent with the literature. For meaningful physical activity, research suggests physical activity to become meaningful when individuals attribute value through lived experience, agency, and intrinsic motivation rather than external outcomes (23, 75, 76). Philosophically, the definition used in the final framework reflects Merleau-Ponty's notion of the “lived body,” positioning meaning as grounded in subjective embodied experience rather than objective measurement (20, 21). Human flourishing was framed as not merely a feeling, but rather an active state of being expressed through action (77). Research highlights that human flourishing involves relational and societal contribution, extending beyond individual wellbeing (78, 79), allowing it to be interpreted through diverse personal values and belief systems while maintaining conceptual clarity. In this paper, human flourishing is conceptualised not as a single outcome or static state, but as a dynamic and context-sensitive process, experienced through lived and embodied interactions with the world, and evidenced through multidimensional wellbeing outcomes. This distinction is important, as it differentiates human flourishing from wellbeing constructs that are often treated as measurable end-states, positioning it instead as an ongoing and relational way of being. For physical literacy, the definition was taken Sport England consensus statement (80), which avoids reducing the concept to discrete components, emphasises integrated experience, and aligns with monist, existential, and phenomenological perspectives, thereby addressing disciplinary variation in interpretation.

Embodiment served as the central integrative mechanism, and was consistently labelled as the “golden thread” anchoring the interconnections between concepts within lived experience. This paper extends existing perspectives by suggesting that physical activity contributes to human flourishing under specific embodied conditions, rather than as a universal outcome of participation. Explicitly, physical literacy may shape an individual's capacity to engage with movement, while meaningful physical activity provides value-laden experiences; however, it is the embodied integration of these experiences that determines whether they contribute to human flourishing.

An important implication for the above perspective is that similar movement behaviours may lead to divergent outcomes. For instance, engagement in the same activity may foster enjoyment, connection, and growth for one individual, while producing disengagement and stress for another. These differences are understood through the embodiment, where personal relevance, prior experiences, and contextual factors shape how movement is perceived and lived (29, 81, 82). By integrating physical literacy, meaningful physical activity, and embodiment, the proposed framework highlights that it is not simply participation in movement that matters, but rather how movement is experienced, interpreted and situated in an individual's life. Recognising the centrality of embodiment offers a more nuanced and practically relevant foundation for promoting human flourishing through physical activity.

Furthermore, by including embodiment, the framework allows for critical reflection on one's conscious awareness of the physical and psychological self, and how they experience the world through senses, thoughts, and actions (17). This aligns with monist philosophical perspectives and emphasises meaning-making through movement (17, 29), thereby supporting vitality, agency, and ultimately, moments of human flourishing through movement. Embodiment therefore represents how the interconnections are enacted in practice.

 In line with Myrto Mavilidi, this research highlights that movement-based approaches are not inherently beneficial, but depend on the degree to which they are meaningfully integrated with the experience (37, 83). In this sense, integration serves as a critical mechanism linking physical literacy and meaningful physical activity with human flourishing. Embodiment may provide a more precise account of how and when movement contributes to human flourishing, thereby addressing a key limitation in existing approaches that prioritise the quantity of movement over experiential quality. In essence, the framework produced in this paper argues that movement becomes effective for physical literacy and human flourishing when it is meaningfully integrated with the person, the task, and the context.

The fidget spinner furthermore demonstrates the concepts as interdependently linked. For example, positive social relationships, enjoyable experiences, appropriate challenges, and personal relevancies in meaningful physical activity and physical literacy have the potential to determine positive emotion, engagement, relationships, meaning and accomplishment for human flourishing (84–86). Human flourishing is seen as grounded in social and personal connections, positivity and resilience (87, 88), which can be enhanced by meaningful physical activity and physical literacy. So, while each concept has its own emphasis and language, together they have potential to form a holistic, interconnected framework for understanding how movement contributes to a fulfilling life.

In addition, the three concepts are demonstrated through the Spinner Model as dynamic and constantly evolving, with implicit ability for one concept to influence the others. For example, meaningful experiences could have the potential to influence motivation, commitment, engagement, and wellbeing; which are all critical for physical literacy (89, 90). Likewise, development of physical literacy could also be critical for enabling rich and meaningful physical activity experiences (91), supporting a reciprocal connection between these concepts. Nevertheless, physical literacy could influence motivation (92), self-esteem (93), physical and psychological competencies (94–96), as well as quality of life (97), all of which have potential to contribute to human flourishing (87, 98, 99).

Importantly, it was argued that there cannot simply be one concept existing without the other if the concepts were to align with the monist approach. In one context for example, participants summarised that those who have physical literacy are more likely to have more meaningful physical activity experiences, and it is those meaningful physical activity experiences that feed into general feelings of human flourishing. There lies plausibility of an association between physical literacy, meaningful physical activity and human flourishing through the ability of physical literacy to contribute meaningful physical activity experiences (100), as well as isolated components of flourishing (101–104). In this sense, physical literacy is seen to ground development, meaningful physical activity is seen to mediate sustained engagement, and human flourishing is seen to elevate the experience towards aspirational outcomes.

Although, it may be argued that human flourishing does not happen in all meaningful physical activity and physical literacy conditions. There can be moments where the concepts may not be in mutually interdependent states due to individualistic tendencies (e.g., personal motivation, autonomy, variability in engagement) and temporal factors. According to Durden-Myers & Whitehead (4), human flourishing is entirely agent-dependent and does not merely happen or occur independently of the individual; it falls on the individual to be proactive in attributing meaning and value for their physical activities. So, if that individual is not intentionally attributing meaning and value for their physical activities, the activity will not always lead to human flourishing. Human flourishing depends heavily on the circumstances in which one lives (e.g., sociopolitical positions and living environments (99); if an individual is not in the appropriate or ideal environment for their physical activity, they are not necessarily going to flourish. It is therefore intended to make clear that although there lie benefits to the interconnection between meaningful physical activity, physical literacy and human flourishing, such benefits may not always occur when applied to practice.

Furthermore, the implementation of a participant-centred approach rather than a prescriptive “one-size-fits-all” model may facilitate in improving outcomes and experiences by focusing on individual needs, preferences and values (95, 96, 105). By offering a discussion tool through the use of interpretive questions, the framework responds to participant feedback regarding the need to address the individualistic and subjective nature of meaningful physical activity, physical literacy and human flourishing. NHS England (106) highlight how using a person-centred approach to involve people in their own care can improve and enhance individual experiences, and potentially yield cost savings through supporting people to manage their health on their own. This framework could be novel in providing early preventative care to communities by teaching them how to explore their own movement values and discover ways to enhance their health and human flourishing in a manner that is suitable for them. By facilitating and supporting individuals to explore their movement autonomously, they may become more inclined to develop intrinsic values that sustain movement and human flourishing longer term (107). Nevertheless, by providing individuals with opportunities for critical reflection and discussion regarding meaningful physical activity, physical literacy and human flourishing, individuals will begin to understand social justice for movement and physical activity, positioning them to have an active role in building a more diverse, inclusive, and equitable society (108). Therefore this framework, while highlighting the interconnectedness between concepts, it provides opportunities for critical thinking into enhancing and enabling high quality movement experiences for all.

To assert the practical utility of the framework, two examples are applied across community, and public health contexts. First, within a community physical activity programme, such as a local “active ageing” walking initiative, sessions could be structured to emphasise autonomy, social connection, and environmental engagement rather than step count, or minutes of walking alone. Facilitators may incorporate flexible walking routes, conversation prompts, and opportunities for participant co-design activities to truly capture meaningful embodied experiences. This aligns with meaningful physical activity principles by foregrounding personal relevance and enjoyment, while supporting embodiment, social relationships, and self-perceived capability. In this context, human flourishing may emerge through sustained participation, social belonging, and increased confidence in everyday movement.

Second, within a public health or clinical referral context (e.g., exercise referral schemes), the framework could guide practitioners to move beyond exercise targets and instead focus on co-creating meaningful and embodied movement experiences that align with an individual's own internal values, capabilities, and lived circumstances. For example, a patient referred for low mood could be supported to identify preferred forms of movement (e.g., dancing, gardening), and encouraged to reflect on how these activities influence mood, agency, and daily functioning. This reflects embodiment, in which movement is experienced as meaningful and integrated within the individuals own context, as opposed to an externally imposed intervention.

These two examples illustrate how physical literacy, meaningful physical activity, embodiment and human flourishing may be operationalised through context-sensitive and participant centred approaches that prioritise the quality of movement experiences. However, further research is required to examine the mechanisms through which embodied, context-sensitive movement experiences influence outcomes, and to identify the conditions under which such approaches support, limit, or fail to promote human flourishing.

### 5.1 Study limitations

The study had limitations. As with many Delphi studies, participant attrition occurred across rounds (109), with one round consisting of only three participants due to scheduling conflicts and competing commitments. Although, small sample sizes can be acceptable in Delphi research (43), this may have impacted the robustness of consensus and interpretation for that round (45). To mitigate this, participants were offered opportunities to take part in follow-up interviews email exchange after reviewing the discussion summary and transcripts for each round. Future should consider strategies to minimise attrition, such as the use of incentives to maintain participation (110).

A further limitation relates to the iterative nature of the Delphi process, where participants are able to revise their views across rounds. While this is a methodological strength, it may also introduce bias, particularly where participants are exposed to alternative or dominating perspectives (111). In the present study, power dynamics were observed, with academics often expressing stronger theoretical positions than practitioners and policy stakeholders. Although follow-up opportunities were provided to encourage independent reflection, this does not fully mitigate the potential influence of such dynamics. Future research may benefit from larger and more diverse samples, as well as profession-stratified focus groups, to reduce the impact of hierarchal influence and support balanced contributions (110, 112).

## 6 Conclusion

Overall, this study presents a dynamic, evolving framework that conceptualises meaningful physical activity, physical literacy, and human flourishing as an integrated system rather than isolated constructs. The revised framework offers concise, evidence-informed, and conceptually aligned definitions of the three concepts, enhancing clarity and accessibility. The refined framework visually demonstrates the interdependent relationships among physical literacy, meaningful physical activity, and human flourishing, providing a tool for understanding and fostering quality movement experiences. Embodiment is positioned as the central “golden thread” connecting the constructs, reflecting lived, phenomenological experiences of movement. The framework also supports critical reflection, allowing stakeholders in research, policy, and practice to interpret and apply the model within their own contexts to promote human flourishing through movement. Overall, the framework provides a robust, flexible, and conceptually coherent tool for understanding and enhancing wellbeing through physical activity.

## Data Availability

The data that support the findings of this study are available from the corresponding author, Olivia Hawes, upon reasonable request.
