# Supplementary material for: The spinner model for flourishing through movement: a conceptual framework for exploring the interplay between physical literacy, meaningful physical activity, embodiment, and human flourishing
Source: Front Sports Act Living. 2026 Jul 10;8:1819295. doi: 10.3389/fspor.2026.1819295 (PMC13395861; doi:10.3389/fspor.2026.1819295)
Supplement: Supplementary file 1 [file Supplementaryfile1.docx]

**Expert Evaluation Form**

Towards Consensus: Using the Delphi Method to Validate a Framework for Meaningful Physical Activity Experiences, Physical Literacy, and Human Flourishing

This form asks you to review the developed framework and rate a series of statements on a five-point scale, from Strongly Disagree to Strongly Agree. It also includes several open-ended questions where you can provide additional comments or suggestions. Your feedback will be used to identify areas where the framework could be clarified, expanded, or refined to better capture the interconnections between meaningful physical activity, physical literacy, and human flourishing. The collective input from all experts will inform the next version of the framework, which will then be discussed in the focus group sessions.

| *Expert Information* | |  |  |  |  |  |
| --- | --- | --- | --- | --- | --- | --- |
| Name |  |  |  |  |  |  |
| Organisation/Institute |  |  |  |  |  |  |
| Position |  |  |  |  |  |  |
| e-mail |  |  |  |  |  |  |
| *Criteria* | | Strongly Disagree | Disagree | Neither Agree nor Disagree | Agree | Strongly Agree |
| The framework provides sufficient information to understand meaningful physical activity experiences, physical literacy, and human flourishing (sufficiency). | |  |  |  |  |  |
| The framework covers the essential elements related to meaningful physical activity experiences, physical literacy, and human flourishing (relevance). | |  |  |  |  |  |
| The information in the developed framework is accurate and can be supported by comprehensive evidence (accuracy). | |  |  |  |  |  |
| The developed framework addresses the interconnectedness between physical literacy, meaningful physical activity, and human flourishing (relevance). | |  |  |  |  |  |
| The developed framework comprehensively covers components essential for promoting human flourishing (comprehensiveness). | |  |  |  |  |  |
| The language and terminology used are clear and understandable (understandability) | |  |  |  |  |  |
| The developed framework is user friendly (ease of use). | |  |  |  |  |  |
| The developed framework is practical for use advancing research, policy, or practice in the field (practicality/usefulness). | |  |  |  |  |  |

Q1. What additional information or details could be included in the framework to improve its overall sufficiency? (sufficiency)

Q2. Are there any sections where you see a need for more supporting evidence to support accuracy? (accuracy)

Q3. In what ways could the framework more effectively address the interconnectedness between meaningful physical activity experiences, physical literacy, and human flourishing? (relevance)

Q4. What additional components, if any, do you think could be considered to enhance he frameworks comprehensiveness? (comprehensiveness)

Q5. Are there specific terms or concepts you feel need additional explanation for the broader audience? (understandability)

Q6. How do you feel the framework could be adapted to ensure user-friendliness? (ease of use)

Q7. What ways could the framework be adapted to increase its practical usefulness for advancing research, policy, or practice? (usefulness/practicality)

Q8. Do you have any considerations for refining the developed framework to make it more feasible for implementation? (practicality)

Q9. Is there anything lacking from the framework? If so, what is lacking, and how can this be improved? (comprehensiveness)
